# Microbiota-Gut-Brain Axis in Age-Related Neurodegenerative Diseases

**DOI:** 10.2174/1570159X23666241101093436

**Published:** 2024-11-04

**Authors:** Tong Nie, Li You, Fang Tang, Yanhui Duan, Eugenie Nepovimova, Kamil Kuca, Qinghua Wu, Wei Wei

**Affiliations:** 1 College of Life Science, Yangtze University, Jingzhou, 434025, China;; 2 College of Physical Education and Health, Chongqing College of International Business and Economics, Chongqing, 401520, China;; 3 College of Humanities and New Media, Yangtze University, Jingzhou, 434025, China;; 4 School of Pharmaceutical Science and Technology, Hangzhou Institute for Advanced Study, University of Chinese Academy of Sciences, Hangzhou, 310024, China;; 5 Department of Chemistry, Faculty of Science, University of Hradec Králové, 500 03, Hradec Králové, Czech Republic;; 6 Biomedical Research Center, University Hospital of Hradec Králové, 500 05, Hradec Králové, Czech Republic;; 7 State Key Laboratory for Managing Biotic and Chemical Threats to The Quality and Safety of Agro-Products, Key Laboratory of Traceability for Agricultural Genetically Modified Organisms, Ministry of Agriculture and Rural Affairs, Zhejiang Academy of Agricultural Sciences, Hangzhou, 310021, China

**Keywords:** Neurodegenerative diseases, gut microbiota, blood-brain barrier, microbiota-gut-brain axis, therapy, dietary adaptations

## Abstract

**Background:**

Age-related neurodegenerative diseases (NDs) pose a formidable challenge to healthcare systems worldwide due to their complex pathogenesis, significant morbidity, and mortality.

**Scope and Approach:**

This comprehensive review aims to elucidate the central role of the microbiota-gut-brain axis (MGBA) in ND pathogenesis. Specifically, it delves into the perturbations within the gut microbiota and its metabolomic landscape, as well as the structural and functional transformations of the gastrointestinal and blood-brain barrier interfaces in ND patients. Additionally, it provides a comprehensive overview of the recent advancements in medicinal and dietary interventions tailored to modulate the MGBA for ND therapy.

**Conclusion:**

Accumulating evidence underscores the pivotal role of the gut microbiota in ND pathogenesis through the MGBA. Dysbiosis of the gut microbiota and associated metabolites instigate structural modifications and augmented permeability of both the gastrointestinal barrier and the blood-brain barrier (BBB). These alterations facilitate the transit of microbial molecules from the gut to the brain *via* neural, endocrine, and immune pathways, potentially contributing to the etiology of NDs. Numerous investigational strategies, encompassing prebiotic and probiotic interventions, pharmaceutical trials, and dietary adaptations, are actively explored to harness the microbiota for ND treatment. This work endeavors to enhance our comprehension of the intricate mechanisms underpinning ND pathogenesis, offering valuable insights for the development of innovative therapeutic modalities targeting these debilitating disorders.

## 1 INTRODUCTION

**Fig. (1):**
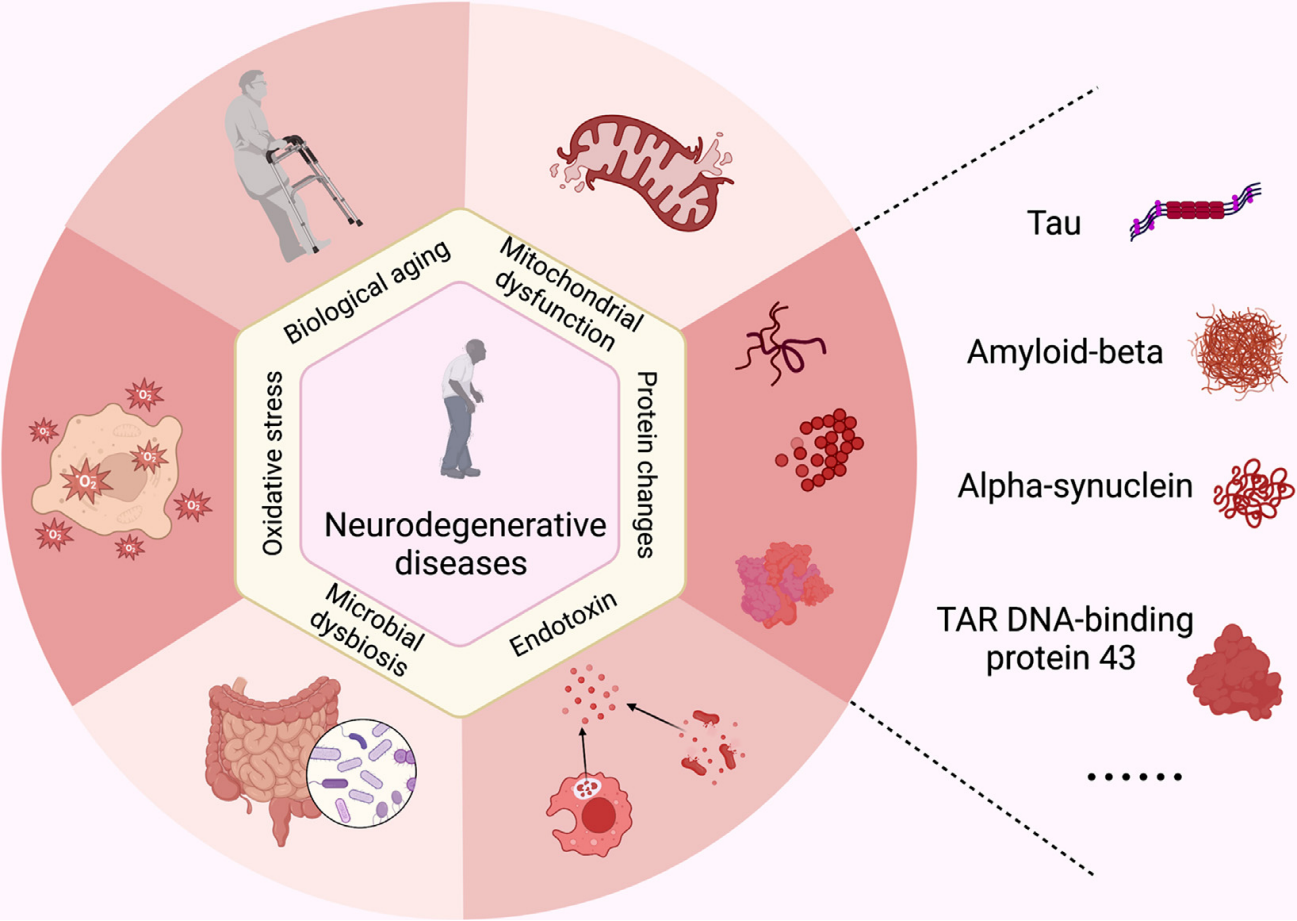
Partial hallmarks of neurodegenerative diseases (NDs). NDs have multiple and complex etiologies, with mitochondrial dysfunction, biological aging, and oxidative stress being closely linked to their development. The misfolding, aggregation, and accumulation of various proteins play a significant role in the development of NDs. For example, in Alzheimer's disease (AD), the accumulation of Amyloid-beta (Aβ) and tau proteins; in Parkinson's disease (PD), multiple sclerosis (MS), and dementia with Lewy bodies, the aggregation of Alpha-synuclein (α-Syn) takes place; in amyotrophic lateral sclerosis (ALS), the accumulation of TAR DNA-binding protein 43 (TDP-43) occurs. The gut endotoxin can be attributed to intestinal permeability, which allows it to enter the bloodstream. Subsequently, the circulating endotoxin can trigger brain inflammation, ultimately leading to the development of NDs. The gut microbiota and its metabolites are also involved in NDs through a continuous and bidirectional communication pathway known as the gut-brain axis. Dysbiosis of the gut microbiota can lead to the impairment of both the gut barrier and the blood-brain barrier (BBB) permeability. The disruption allows for the development of diseases through various routes, including neural, endocrine, and immune routes.

Neurodegenerative diseases (NDs) are disorders characterized by the chronic and progressive loss of neuronal structure and function. The incidence of NDs shows a positive correlation with the rapidly aging population, leading to a growing burden on society [1, 2]. Currently, there is a large amount of attention on NDs, including Alzheimer's Disease (AD), Parkinson's Disease (PD), Huntington's Disease (HD), Multiple Sclerosis (MS), and Amyotrophic Lateral Sclerosis (ALS) [3, 4]. These diseases share common characteristics, such as a higher incidence in older individuals, neuronal damage in the brain, disruption of synaptic connectivity networks, and selective loss of brain mass [5]. In previous studies, abnormal tau aggregates [6], amyloid-β oligomer (AβO) [7], and mitochondrial dysfunction [8] were shown to be associated with NDs. Additionally, protein misfolding, aggregation, and accumulation in the brain have been identified as potential causes of these diseases, leading to neuronal apoptosis, loss of synaptic connections, and brain damage [9]. Naturally, different protein aggregates are implicated in specific NDs. For instance, alpha-synuclein (α-Syn) aggregates are associated with PD, MS, and dementia in Lewy bodies. TAR DNA-binding protein 43 (TDP-43) accumulates in ALS, while amyloid-beta (Aβ) and tau are associated with AD [10]. In addition, there is increasing evidence suggesting that endotoxins may act as triggers for NDs, potentially synergizing with various protein aggregations [11] (Fig. **1**).

Most recently, increasing lines of evidence have suggested that the microbiota-gut-brain axis (MGBA) is involved in the pathogenesis of NDs [2, 12, 13]. The gut microbiota interacts with the brain by a continuous and bidirectional gut-brain axis [14]. Dysbiosis of the gut microbiota disrupts the integrity of the gut barrier and the blood-brain barrier (BBB), which can lead to the development of various pathologies *via* the neural, endocrine, and immune routes [15, 16]. The gut microbiota, originating from various sources, produces various metabolites, including trimethylamines (TMAs), short-chain fatty acids (SCFAs), and vitamins [2]. The gut microbiota and its metabolites, such as probiotics, prebiotics, and dietary nutrients, may provide potential therapeutic targets through the gut-brain axis for delaying the onset, slowing down the progression, or even reversing NDs [17, 18]. Nonetheless, we consider that there are still many uncertainties surrounding the MGBA that require further clarification. For instance, it is not yet fully understood what specific changes occur in the gut barrier and the BBB in patients with ND. Additionally, the impact of numerous gut microbiota and its metabolites on the multiple routes of the MGBA remains to be determined. Lastly, the effectiveness of MGBA-targeted therapies in treating NDs still needs to be investigated. These uncertainties compel us to delve deeper into the emerging field of the MGBA, approaching it with fresh perspectives and striving to make new advancements in research related to NDs.

In this review, we aim to provide a comprehensive overview of the recent research advancements that have contributed to the elucidation of the mechanisms underlying the role of the gut microbiota, particularly the MGBA, in NDs. In addition to summarizing the programmatic strategies for treating ND through modulation of the gut microbiota, we further discuss the current challenges in this field and look forward to where this field goes. This work helps to enhance our understanding of the pathogenic mechanisms underlying NDs and propel the development of clinical treatment modalities.

## 2 THE GUT MICROBIOTA AND ITS METABOLITES IN NDs

At present, part of the investigations have demonstrated that certain species of the gut microbiota and its metabolites are strongly associated with NDs [19]. The gut microbiota, comprised of bacteria, viruses, and fungi, plays a significant role in the gastrointestinal tract by engaging in various essential chemical processes that produce a diverse range of metabolites [20]. Based on the modification performed by the gut microbiota on different substrates, these metabolites can be broadly categorized into three groups: metabolites that are directly produced by the gut microbiota from the diets, metabolites generated by the host but modified by the gut microbiota, and metabolites that are formed *de novo* [21, 22]. These metabolites encompass a range of compounds such as short-chain fatty acids (SCFAs), Lipopolysaccharide (LPS), bile acids, derivatives of tryptophan and indole, choline metabolites, neurotransmitters, and lipids [23]. Not all of the gut microbiota and its metabolites have been fully understood in NDs so far. However, there are at least 19 gut microbiota traits that have been identified as causally associated with NDs, and 12 gut microbial metabolites with suggestive associations with NDs [24]. For example, the genetically predicted abundance of *Ruminococcus* is positively correlated with the risk of ALS, and a reduction in the metabolite glutamine is also associated with AD, while 5-hydroxytryptamine (5-HT) is protective against PD and kynurenine is a risk factor for ALS [24]. In addition, the concentration levels of the most abundant microbial metabolites, SCFAs, are closely associated with mild cognitive impairment, AD, and PD [25, 26]. That is, a wide range of microorganisms in the gut build a pathological association with NDs.

**Fig. (2):**
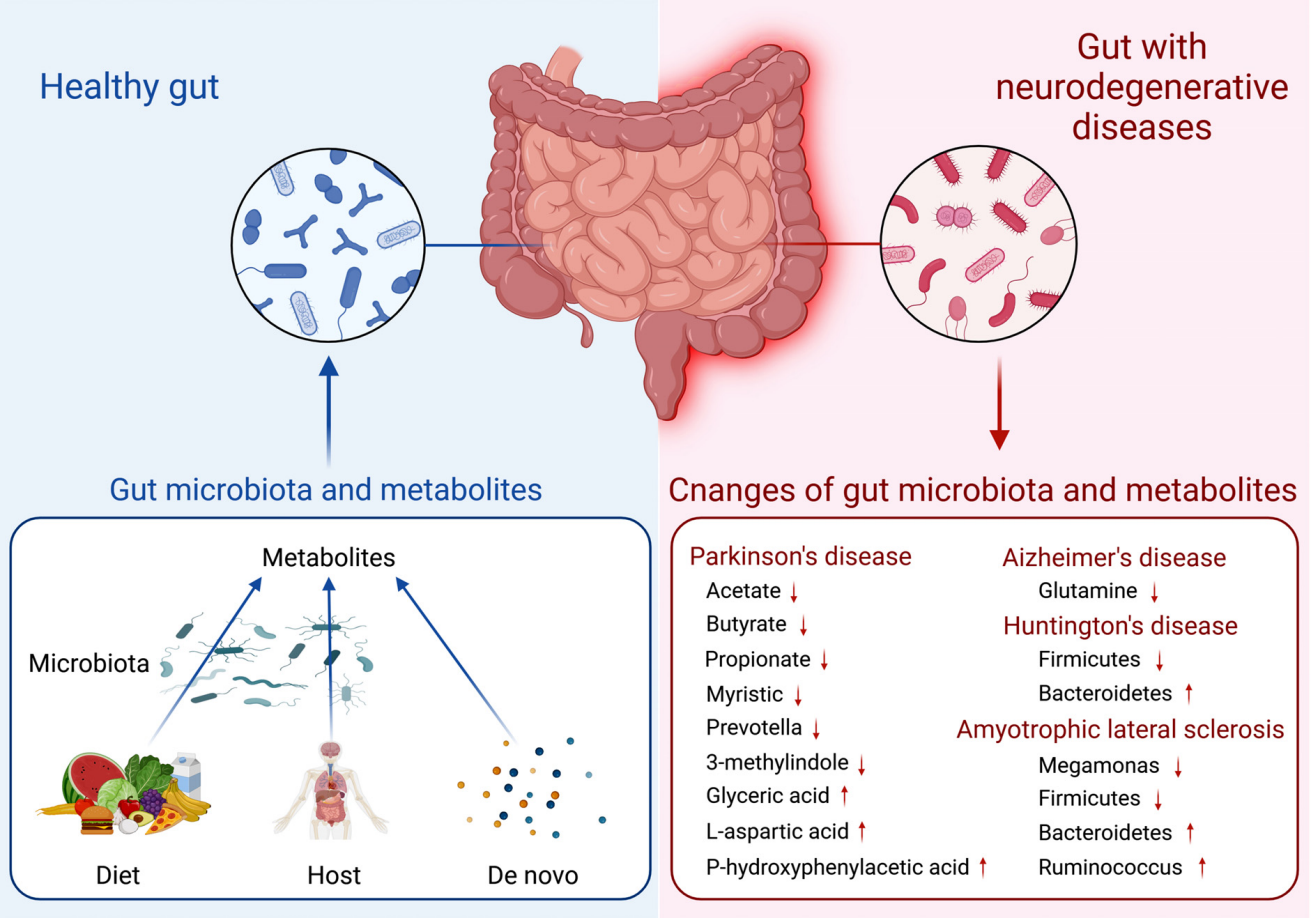
Alterations in the gut microbiota and its metabolites in patients with neurodegenerative diseases (NDs). In a healthy gut, the metabolites present are primarily produced by the gut microbiota directly from dietary sources or generated by the host and subsequently modified by the gut microbiota or formed *de novo*. However, in patients with NDs, the gut microbiota and its metabolites undergo distinct alterations due to a combination of complex internal and external factors, including host genetic predisposition, environmental factors, lifestyle, diet, and the use of antibiotics and other medications. Nonetheless, targeting the gut microbiota through interventions such as fecal microbiota transplantation (FMT), product interventions, and dietary modifications has shown promise in improving the symptoms and progression of NDs.

Alterations in the gut microbiota and its metabolites are common in NDs, but these alterations vary between different types of diseases [19] (Table **1**). An analysis of the gut microbiota and its metabolites in A53T transgenic monkeys with early Parkinson's symptoms showed a significant decrease in *Prevotella* and an increase in the metabolites glyceric acid, L-aspartic acid, and p-hydroxyphenylacetic acid, and a decrease in myristic acid and 3-methylindole [27]. The stool specimens from individuals with PD reveal that the levels of SCFAs, namely acetate, butyrate, and propionate, exhibit a marked decrease, with a particularly notable reduction observed in propionate [28]. In the R6/1 transgenic mouse model of HD, there is an increase in *Bacteriodetes* and a proportional decrease in *Firmicutes* [29]. Furthermore, a study of the diversity of the fecal community in ALS patients showed that *Bacteroidetes* were highly expressed at the phylum level and several microbes at the genus level. In contrast, *Firmicutes* at the phylum level and *Megamonas* at the genus level were highly expressed in healthy individuals [30]. As can be seen from these cases, alterations may be present in other NDs as well. The gut microbiota and its metabolites are so enormous and diverse that it is impractical to completely count all the different types that currently exist. Meanwhile, the colonization of the gut microbiota in individuals can be influenced by a variety of factors, including genetic predisposition, environmental factors, lifestyle, diet, and antibiotic and non-antibiotic drug use, resulting in wide variation [20, 31]. Accordingly, we consider that the alterations in the gut microbiota and its metabolites in the various NDs shown above are common rather than comprehensive (Fig. **2**).

Fecal microbiota transplantation (FMT) can be used to verify the alterations that are caused by the dysbiosis of the gut microbiota [32, 33]. FMT reconstructs the gut microbiota by transplanting functional fecal microbiota from a healthy individual to one with NDs [34]. For example, it can ameliorate gut dysbiosis and cognitive deficits in HD mice and appears to alleviate constipation symptoms in Parkinson's patients [35, 36]. However, reversing the whole process has also given us insight into the consequences of gut microbial dysbiosis. Transferring microbiota from old to young mice accelerates age-associated central nervous system (CNS) inflammation and increases gut barrier permeability, whereas transferring young donor microbiota can reverse these detrimental effects [33]. In addition, FMT of 5xFAD (transgenic mice with five familial Alzheimer's disease) mice into normal C57BL/6 mice decreases hippocampal neurogenesis and brain-derived neurotrophic factor expression and increases p21 expression, leading to memory impairment [32]. Moreover, activated microglia result in an increase in pro-inflammatory cytokines, including tumor necrosis factor-alpha (TNF-α) and interleukin-1 β (IL-1 β), as well as increased pro-inflammatory cytokines in the colon and plasma [32]. This means that dysbiosis of the gut microbiota reduces neurogenesis by increasing inflammation, leading to memory loss. We regard FMT not only as a means to explore the role of microbial dysbiosis in relation to NDs but also as a potential therapeutic option.

The gut microbiota and its metabolites develop specific linkages with the pathology of NDs. Alterations of these microorganisms in the gut are one of the factors in triggering NDs. In different types of NDs, the gut microbiota and its metabolites present different changes, with some increasing and some decreasing. However, we are currently unable to fully enumerate the changes in gut microorganisms that occur in NDs since they are highly individual-specific. This hinders the exploration of the gut microbiota and its metabolites in NDs. During the exploration, FMT is regarded as a method to validate that gut dysbiosis contributes to NDs, which can yield powerful supporting evidence. FMT is also used as one of the strategies to treat NDs by improving the status of gut microbiota and its metabolites. Taken together, we believe that gut microbiota and its metabolites are excellent candidates for deeply investigating NDs.

## 3 THE DYSFUNCTION OF GUT BARRIER IN NDs

There is a dysfunction of the gut barrier in NDs following microbiota dysbiosis. NDs are associated with gut microbiota dysbiosis [29, 37, 38], which is characterized by an overall reduction in microbiota diversity and a marked change in its composition [39]. Moreover, it is always associated with gut barrier dysfunction [40]. The leaky gut barrier in patients with ND exhibits mucus degradation in the luminal mucus layer [40], which means a decrease in mucus glycosylation levels and allows widespread microbiota to penetrate the luminal mucosal layer [41]. With progressive damage to the gut barrier, the expression of the junctional protein complexes between gut epithelial cells, consisting of occludin, E-cadherin and Zonula occludens protein 1 (ZO-1) is reduced and the cells gradually become sparsely arranged [41, 42]. It allows the unrestricted crossing of microbiota and metabolites, and then the lymphocytes gradually increase, initiating the immunoregulatory processes [42, 43]. Of note, the gut microbiota can cause dysfunction, and the disruption of the gut barrier can also influence the microbiota [44]. It implies that a feedback loop developed between them will continually deteriorate the healthy condition of the gut.

Dysfunction of the gut barrier is primarily reflected in increased permeability associated with intestinal and systemic inflammation [45, 46]. As permeability increases, the microbial molecules can enter the circulation unrestricted through the leaky gut, contributing to the pathogenesis of NDs [43]. There is the case of *Candida albicans*, which is one of the most common commensal fungi of the human gut. Its individual cells and cell clusters were discovered in or near the vessels, ventricular spaces, cerebellum, hypothalamus, midbrain, and cortex of C57BL/6 mice [47]. This indicates that *Candida albicans* can translocate from the gut to the brain *via* circulation in model mice and cause gut, brain, and systemic inflammatory responses that exacerbate the development of ND. The presence of inflammatory responses can be demonstrated by detecting the expression levels of inflammatory markers in mouse models induced by paraquat (a potential environmental risk factor for NDs) [42]. These markers show varying degrees of high expression in the Lewy body disease time-series model mice exposed to paraquat [42]. Namely, the increased permeability enables gut microbiota and its metabolites to cross the gut and then induce inflammatory responses at multiple sites.

**Fig. (3):**
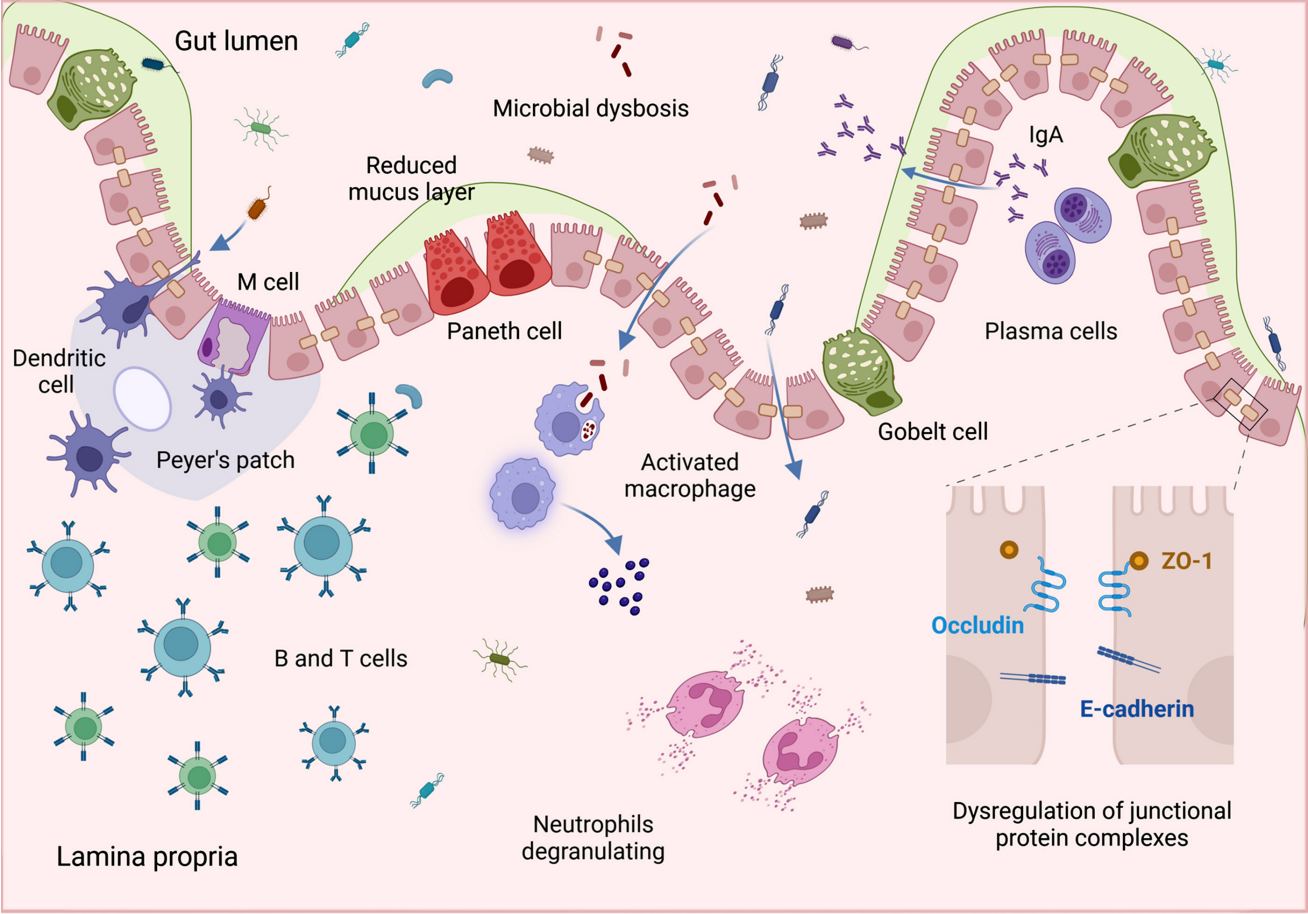
Impaired gut barrier in patients with neurodegenerative diseases (NDs). In NDs, there is an overall reduction in the diversity of the gut microbiota and significant alterations in its composition, a condition known as microbial dysbiosis, which is often accompanied by dysfunction of the gut barrier. The dysfunction of the gut barrier in NDs primarily manifests in several ways. It included degradation of the luminal mucus layer, a reduction in the expression of junctional protein complexes such as occludin, E-cadherin, and Zonula occludens protein 1 (ZO-1) between the gut epithelial cells, a gradual disruption in cell arrangement, and an increase in permeability. These dysfunctions enable the unrestricted passage of microbial molecules through the leaky gut, leading to a progressive increase in lymphocytes and the initiation of immune regulatory processes. This, in turn, triggers inflammation both within the gut and systemically. Therefore, the dysbiosis of the gut microbiota, dysfunction of the gut barrier, and inflammation collectively contribute to the development of NDs.

Gut microbiota dysbiosis, gut barrier dysfunction, and inflammation combine to drive the development of NDs. A single “pro-inflammatory factor” does not influence ND patients. There are no significant changes in tight junction proteins in R6/2 mice at 12 and 16 weeks of age, but they still display an increase in microbiota dysbiosis and gut permeability, exacerbating HD [48]. This could be due to differences in the model mice or to instantaneous changes in tight junction proteins at a particular stage. The authors could add different models or test whether there are instantaneous changes to confirm the previous ideas. Alcohol-induced gut microbiota dysbiosis and increased gut permeability are not sufficient to exacerbate disease in rodent models of AD [49]. In addition, gut barrier disruption without apparent gut inflammation is not associated with exacerbation of PD-like behavior and pathology [44]. This may also be due to the absence of systemic inflammation. As a result, gut microbiota dysbiosis and gut barrier dysfunction may typically occur together, with inflammation being the primary driver mediating the progression of NDs [44]. Thus, we strongly believe that gut microbiota dysbiosis, gut barrier dysfunction, and systemic inflammation are among the key factors contributing to ND in patients (Fig. **3**).

We conclude that the gut barrier is the first gatekeeper, and when it becomes dysfunctional, it can contribute to the development of NDs. Changes in the microstructure of the gut barrier result in increased gut permeability, which allows dysbiotic microbiota and metabolites to pass through and trigger a series of inflammatory responses in the body. These processes are demonstrated to drive the pathological progression of NDs while significantly elevating the plausibility that MGBA mediates NDs. Furthermore, these also implicitly point to the suppression of gut microbiota dysbiosis, gut barrier dysfunction, and inflammatory responses as potential therapeutic strategies for NDs, favoring disease control.

## 4 DYSFUNCTION OF THE BLOOD-BRAIN BARRIER IN NDs

Current evidence indicates that gut microbiota-derived metabolites have an impact on the integrity and function of the blood-brain barrier (BBB) [50, 51]. The BBB is influenced by gut microbiota-derived metabolites, as well as factors in the brain, such as restraint stress, hormones, and proteins [52-54]. LPS acting on brain endothelial cells can prime *Casp11* and *Cd14* expression and induce the pore-forming protein gasdermin D-mediated plasma membrane permeabilization and pyroptosis [55]. This leads to ultrastructural changes in the BBB, such as pyroptotic endothelia, abnormal appearance of tight junctions, and vasculature detachment from the basement membrane [55]. SCFAs acting on their own or in combinations can modulate select microglial functions [56]. For example, the SCFA mixture including acetate, propionate, butyrate, formate, and valerate, as well as several individual SCFAs, reduce the release of IL-1β, TNF-α, monocyte chemoattractant protein (MCP)-1, and cytotoxins by immune-stimulated microglia-like cells [56]. Formate and valerate, when applied independently, diminish the phagocytic activity; moreover, formate, when acting in isolation, also restricts the activity of respiratory bursts, thereby curtailing the generation of ROS [56]. In addition, propionate and butyrate can induce distinct changes to filamentous actin directionality, increase tight junction protein spikes, protect from LPS-induced tight-junction mislocalization, and finally improve BBB integrity [57]. Propionate also suppresses pathways related to non-specific microbial infection through a CD14-dependent mechanism, inhibits the expression of low-density lipoprotein (LDL)-related protein-1 (LRP-1), and protects the BBB from oxidative stress through NRF2 (NFE2L2) signaling [58]. The metabolites of epicatechin could simultaneously regulate the expression of protein-coding non-coding genes and proteins and reduce permeability that is increased by lipotoxic stress [59]. The derived metabolites of isoflavones and lignans provide protection against neuroinflammatory stimuli in murine BV-2 microglia [60]. Moreover, in the brain, resistin inhibits Toll-like receptor 4 (TLR4)-mediated astrocyte differentiation and alters the microenvironment of neural stem cells [61]. Activated bradykinin 1 receptor (B1R) in the presence of restraint stress influences the cytokine profile and junctional properties of microvascular endothelial cells [52]. Myosin II-mediated contractility is negatively correlated with ZO-1 presentation [62]. CD147 and syndapin-2 are associated with imbalances in overall Aβ clearance, BBB maintenance, and transport during AD pathology [63, 64]. Expansion of CAG repeats alters the trajectory of brain microvascular endothelial-like cell differentiation [65]. TDP-43 mediates permeability and leukocyte infiltration and promotes neurodegeneration in a mouse model with a persistently elevated systemic inflammatory state [66]. Simply put, there is a phenomenon of BBB dysfunction in NDs caused by gut microorganisms.

Dysfunction of the BBB occurs in patients with ND and is characterized by various changes in BBB cell populations [67, 68]. The intact BBB plays a crucial role in regulating the transport of circulating energy metabolites and essential nutrients, controlling cerebral blood flow and permeability, and facilitating the clearance of substances [69]. The BBB cell populations consist mainly of endothelial cells, endothelial cell-sealing tight junction proteins, pericytes, and astrocyte end-feet [67]. In the dysfunctional BBB, the brain microvascular endothelial cells exhibit ultrastructural changes, along with alterations in protein expression of tight junctions, adherens junctions, and glucose transporter type 1 (GLUT-1) [53], resulting in disorders of barrier maintenance and transporter regulation. Pericytes, which are located between brain capillary endothelial cells, astrocytes, and neurons, develop in a degenerate manner or are damaged or even lost [70-72]. This could lead to dysfunction of contractile pericytes, resulting in changes in blood flow, white matter dysfunction, and a neurodegeneration cascade [70-72]. In astrocyte end-feet, a significant increase in aquaporin 4 (AQP-4) was observed, indicating an alteration in astrocytes [53]. Astrocytes and microglia can be activated, and both are transformed into cells with a reactive phenotype [66, 73]. Subsequently, neurons appear with damage, loss of connections, synaptic dysfunction, which ultimately leads to NDs [73]. These findings above indicate that altered BBB cell populations and dysfunction are part of the initiating signs of brain lesions in patients with NDs.

**Fig. (4):**
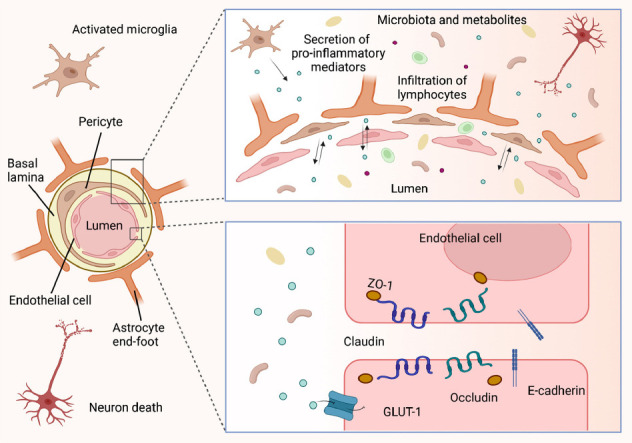
Compromised blood-brain barrier (BBB) integrity in patients with neurodegenerative diseases (NDs). In patients with NDs, the cell population comprising the BBB undergoes a series of alterations. Brain microvascular endothelial cells exhibit ultrastructural changes. Pericytes can undergo degeneration, damage, or even loss, while astrocytes and microglia may become activated and transform into cells with a reactive phenotype. There are notable changes in the localization and expression of key proteins involved in maintaining the integrity of BBB, such as Zonula occludens protein 1 (ZO-1), occludin, claudin-5, and GLUT-1. There is an increase in gaps at tight junctions, disruption in the arrangement of the cytoskeleton, a decline in pericyte coverage, and damage and thickening of the basal membranes. These alterations lead to a progressive increase in the permeability of the BBB, allowing the gut microbiota and its metabolites to cross the barrier. This, in turn, triggers inflammatory and immune responses. Neurons may experience injury, synaptic dysfunction, and loss of connectivity, all of which contribute to the development of NDs.

Similar to gut barrier dysbiosis, BBB dysfunction is characterized by increased permeability and inflammatory responses [74]. The dysfunctional BBB exhibits damaged and thickened basement membranes [53], disrupted cytoskeletal organization [75], and increased gaps in cell junctions [53, 76]. There is decreased expression of junction-related proteins, including ZO-1, occludin, claudin-5, and von Willebrand factor (vWF) [63, 77], and decreased pericyte coverage [70]. These changes ultimately lead to an increase in BBB permeability. With increased permeability, there is a leakage of blood-derived molecules and peripheral cells from the vessels, a decrease in cerebral blood flow [71], and a reduction in the diameter and branching of blood vessels [78]. At the same time, the disrupted BBB allows inflammatory cells, soluble neurotoxic proteins, and pathogens to penetrate the brain, as well as imbalances in the clearance of proteins associated with NDs, such as Aβ [73, 77]. As a result, these persistent disruptions facilitate leukocyte infiltration into the brain and increase the number of lymphocytes and macrophages in the brain, thereby activating a variety of inflammatory and immune responses [73]. That is, both the increased permeability and inflammatory responses of the BBB have destructive effects on glial cells and neurons, ultimately contributing to the development of NDs (Fig. **4**).

Therefore, we can intuitively understand that gut microbiota-derived metabolites affect the BBB. Gut microorganisms disrupt the structural integrity of the BBB and then lead to BBB dysfunction. The increased permeability and inflammatory responses damage neurons, promoting the progression of NDs. These processes still prove the existence of the MGBA and the fact that the gut microbiota and its metabolites induce NDs. Simultaneously, some new issues have come to light. For example, SCFAs have protective effects on the BBB [56-58], but the specific types and exact concentrations of the associated effects are not yet fully known. The metabolites of epicatechin, isoflavones, and lignans may protect the BBB [59, 60], but whether they have destructive effects on the BBB requires further investigation. We need more details about the gut microbiota and its metabolites to confirm their precise effects on NDs. Currently, we realize that gut microbiota dysbiosis triggers gut barrier dysfunction, gut and systemic inflammation, and then BBB dysfunction. All of these together promote neurodegeneration during the pathological process of NDs. However, we are just roughly aware that the gut microbiota and its metabolites eventually reach the BBB through the gut-brain axis, so how they are transferred to the brain should be further explored.

## 5 ROLE OF THE MICROBIOTA-GUT-BRAIN AXIS (MGBA) IN NDs

The MGBA, considered a bidirectional interaction, is a complex communication system, and its bottom-up and top-down regulatory axis represents the gut microbiota and the CNS that can be mutually influenced by neural, hormonal, and immune signals [14]. Currently, the MGBA is not fully understood but is known to integrate extrinsic sympathetic and parasympathetic branches of the autonomic nervous system (ANS), particularly the vagal nerve [79], intrinsic branches of the enteric nervous system (ENS), and the hypothalamic-pituitary-adrenal (HPA) axis [80, 81]. The gut microbiota and its metabolites can induce gut barrier and BBB dysfunction *via* neural, endocrine, and immune routes or a cross combination and subsequently promote neurodegeneration [82]. On the other hand, the nervous system exposed to abnormal emotional factors, traumatic brain injury, and chronic CNS bacterial infections can also alter the composition and function of the gut microbiota [83-88]. A closed loop exists between the CNS and the gut microbiota through the gut-brain axis, bridging them together. Thus, an adequate understanding of these routes will facilitate the identification of potential therapeutic targets for NDs.

The gut microbiota and its metabolites can rapidly communicate with the brain through the vagal nerve. Glutamate can transmit gut sensory signals to the brain *via* the vagal nerve [89], and Aβ can also travel from the gastrointestinal tract to the brain *via* the vagal nerve to induce cognitive impairment [90]. These neuroactive metabolites and neurotransmitters of the gut microbiota, including glutamate, acetylcholine, 5-HT, and gamma-aminobutyric acid (GABA), can participate in the vagal route in various ways [89, 91, 92]. Some of the neuroactive metabolites and neurotransmitters can activate enteroendocrine cells (EECs) to release the neurotransmitter 5-HT to directly stimulate the vagal sensory ganglia. A proportion can stimulate the neuropod cells, the EECs that synapse with vagal neurons, and then transmit transcriptional signals of neuroactive metabolites to the vagal nerve within milliseconds. Some can also enter the gut through the broken gut barrier and make direct contact with the vagal nerve, activating the vagal nerve either through specific receptors or more direct routes [92, 93]. For example, cysteine proteases from human commensal bacteria increase the excitability of mouse vagal afferent neurons by activating protease-activated receptor 2 and regulating the voltage dependence of Na^+^ conductance activation [94]. LPS increased the excitability of nodose ganglia neurons *via* TLR4-dependent activation of nuclear factor kappa B (NF-kB) [94]. Accordingly, the vagal nerve receives a variety of gut signals through direct recognition or indirect routes mediated by EECs and then transmits them to the brain, ultimately mediating NDs, and we consider the vagal nerve an important target for the treatment of NDs.

Of course, some microbiota metabolites also communicate with the brain through the neuroendocrine route, similar to the endocrine route [95]. As mentioned in previous sections, the permeability of the gut barrier is gradually increased in patients with ND, allowing the gut microbiota and its metabolites to pass directly through the gut or leaky gut. They then enter the bloodstream through the disrupted BBB and act directly or indirectly on the brain to promote the progression of ND [2, 56]. In addition, the HPA axis is also important in the endocrine route [80, 95]. There is dysregulation of the HPA axis affected by stress and other stimuli, leading to increased production of glucocorticoids (GCs) coupled with hyperactivation of the GC receptor [96, 97]. This is associated with dysbiosis of the gut microbiota and neuroinflammation, leading to cognitive impairment and aggravation of ND symptoms [96, 98]. Furthermore, the likelihood of developing cognitive impairment increases with increased cortisol (one of the GCs) reactivity [99]. The microbiota also affects the various structures (hypothalamus, pituitary, and adrenal glands) of the HPA axis [100]. The gut microbiota and its metabolites can regulate the expression of the gene encoding the CRH receptor type 1 (Crhr1), the Fkbp5 gene, and other hormone-related genes in these structures, thereby influencing the response to acute restraint stress [100]. The mechanisms through which the gut microbiota influence the brain *via* the endocrine route are complex, and further research is required to reveal the exact etiology.

Another essential communication route of the MGBA is the immune route, with inflammatory cytokines running all the way through. The ND patients and mice have a distinct inflammatory cytokine profile both at the intestinal, systemic, and brain levels [32, 101]. The immune cells present in the gut mucosa are stimulated either directly by the gut microbiota or by the production of mediators that affect the enzymes or stimulate the pattern recognition receptors (PRRs) expressed on the immune cells, thereby shaping the immune response [102]. Two of the four major subfamilies of PRRs, the TLRs and the nucleotide-binding oligomerisation domain leucine-rich repeat-containing receptors (NLRs), are currently known to be involved in the pathogenesis of neurodevelopment and NDs *via* the gut-brain axis [103]. For example, the NOD-like receptor protein 3 (NLRP3) inflammasome can coordinate physiology and regulate peripheral and central inflammatory responses in neurological disorders. Furthermore, increased NLRP3 inflammasome in the gut is positively associated with increased astrogliosis and microglial activation, as well as increased NLRP3 inflammasome activation in the brain [104]. TLR4-mediated inflammation also plays an essential role in gut and brain inflammation [105, 106]. Naturally, the immune route can also be regulated downwards along the MGBA. The cholinergic anti-inflammatory pathway is a key way in which CNS modulates peripheral immune homeostasis, particularly in the gut, and its dysfunction is a possible underlying route for gut microbiota dysbiosis in AD [38]. The gut microbiota and its metabolites may influence NDs *via* the immune route, but whether they have a protective role *via* this route remains to be explored.

**Fig. (5):**
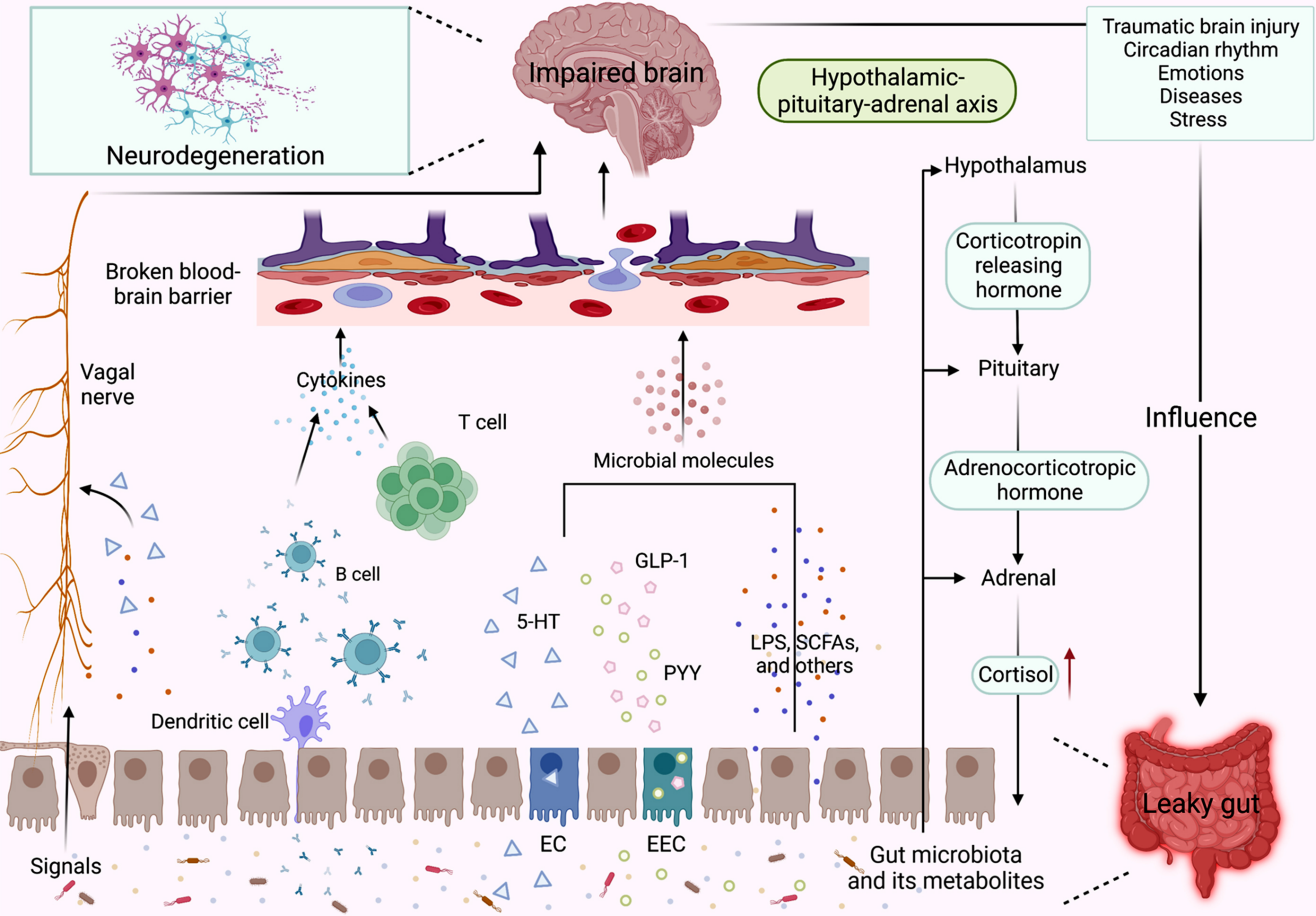
Role of the microbiota-gut-brain axis (MGBA) in neurodegenerative diseases (NDs). The gut microbiota and its metabolites have the ability to communicate with the brain through the vagal nerve. The vagal nerve receives a variety of signals from the gut, including glutamate, acetylcholine, and dopamine, either through direct recognition or indirectly *via* enteroendocrine cells (EECs) and enterochromaffin cells (ECs). These signals are then transmitted to the brain. Another key route is the immune system, whereby inflammatory cytokines are expressed in the gut, brain, and throughout the body. Additionally, the endocrine and neuroendocrine routes play crucial roles in communication between the gut and the brain. Certain microbial molecules, including short-chain fatty acids (SCFAs), 5-hydroxytryptamine (5-HT), lipopolysaccharides (LPS), glucagon-like peptide-1 (GLP-1), and peptide tyrosine-tyrosine (PYY), have the ability to cross the gut or leaky gut, and penetrate the broken blood-brain barrier *via* the circulatory system. These molecules can exert direct or indirect effects on the brain. Likewise, the hypothalamic-pituitary-adrenal (HPA) axis can become dysregulated in response to certain stimuli, leading to increased cortisol responsiveness and impacting the gut microbiota. In turn, the gut microbiota can influence HPA axis. Moreover, exposure of the central nervous system (CNS) to abnormal factors such as emotions, stress, and diseases can also alter the composition and function of the gut microbiota. Therefore, the MGBA forms a closed loop, interconnecting the CNS and the gut.

The dysregulated gut microbiota, leaky gut, and disrupted BBB are closely linked through the neural, immune, and endocrine routes of the MGBA and their cross-talk. The vagal nerve provides more direct communication, allowing the brain and gut microbiota to rapidly detect changes on either side and then modulate accordingly. The endocrine route tends to intersect with the other two routes, which makes it more complicated. The immune route is widespread, which coincidentally demonstrates the presence of gut inflammation, systemic inflammation and neuroinflammation in patients with ND. However, there are still questions to be answered about the MGBA routes. It is not yet entirely clear whether the gut microbiota has a damaging or protective effect on the brain through these routes. In addition to these three basic routes, it is still unclear whether there are other routes by which gut microbiota reach the brain and cause NDs. What's more, based on the key points of these routes, it is currently of interest to test targeted therapeutic routes to suppress the symptoms of the lesion. Therefore, understanding more about the MGBA is of paramount importance for NDs (Fig. **5**).

## 6 TARGETING MGBA FOR THE THERAPY OF NDs

At present, microbiota-based therapies are a way to treat a range of diseases, particularly those that are intractable with limited treatment options [107]. As inspired by FMT, supplementation with pro-, pre-, syn- and post-biotics appears to be a viable method of treating NDs. Probiotics are a group of additional active microorganisms that are beneficial to the host [108]. Prebiotics are substrates that are selectively utilized by gut microbiota, promoting the growth and proliferation of probiotics [109]. Synbiotics refer to the synergistic combination of prebiotics and probiotics, which provide benefits to gut health, metabolism, and the immune system by promoting the survival of gut microbiota [110]. Postbiotics are products derived from non-living microorganisms and/or their constituents [111]. In terms of NDs, postbiotics can mediate neuroprotection *via* increasing the level of dopamine, decreasing the level of α-Syn in substantia nigra, protecting the loss of dopaminergic neurons, reducing the aggregation of neurofibrillary tangles, reducing the deposition of Aβ plagues and ameliorating motor deficits [112]. Furthermore, postbiotics exhibit neuroprotective properties through the suppression of inflammatory responses, inhibition of apoptotic mechanisms, and the enhancement of brain-derived neurotrophic factor secretion [112]. It is noteworthy that various biotic agents, including pro-, pre-, syn-, and post-biotics, exert beneficial effects on the gut microbiota and neurodegenerative diseases through distinct mechanisms [20].

For example, prebiotic mannan oligosaccharide (MOS) and yeast β-glucans could both significantly shape the gut microbiota and enhance the production of neuroprotective metabolites SCFAs [113, 114]. The prebiotic neoagarotetraose (NAT) has been shown to suppress tight junction protein degradation, attenuate inflammatory responses, and reduce injury to cerebral neurons [115]. In particular, NAT treatment could reshape the gut microbiota, regulate the SCFA receptor-related pathway, attenuate Aβ and tau pathology, and ameliorate cognitive impairment in AD mice [115]. Oral administration of *Lactobacillus casei* LC122 or *Bifidobacterium longum* BL986 to aged mice improves their learning and memory abilities and upregulates the expression of neurodegenerative and neurotrophic factors in the hippocampus, including essential secreted proteins [116]. The probiotic mixture of *L. helveticus* R0052 and *B. longum* R0175 can reduce LPS-induced apoptosis in rat hippocampus *via* the gut-brain axis and exert beneficial effects on NDs [117]. The synbiotic supplementation of oligofructose + probiotic for 24 weeks mildly reduces depressive symptoms and improves cognitive functioning in aged people [118]. The administration of synbiotics (*Lactobacillus paracasei* HII01 + *Bifidobacterium animalis* subsp. Lactis + galacto-oligosaccharides + oligofructose) can primarily modulate the activation of HPA-axis and production of IL-10, IgA, and LPS to reduce negative emotions in stressed individuals [119]. Postbiotics include but are not limited to, lactic acid, proteins, vitamins, and SCFAs, as well as pili and cell wall constituents [111, 120], all of which are potential therapeutic agents for NDs. Among them, butyrate is considered a postbiotic with a large therapeutic profile, which contributes to the maintenance of intestinal epithelium and blood-brain barrier integrity, gut motility and transit, anti-inflammatory effects, autophagy induction, and neuronal function [121]. To illustrate with precision, butyrate is capable of mitigating the decrease in BDNF prompted by quinolinic acid through the epigenetic augmentation of H3K18ac at BDNF promoter sites. Moreover, it can reverse neuronal spine degradation and cognitive decline, thereby ameliorating neurodegenerative processes [122]. Accordingly, a variety of products linked to gut microbiota and metabolites could potentially exert neuroprotective effects *via* diverse mechanisms.

In addition to these, some medicines or other naturally derived substances have been found to be effective in treating NDs. Examples include some Chinese herbal medicine formulas. Jiao-tai-wan may alleviate systemic inflammation and cognitive impairment by preventing the transfer of inflammatory triggers *via* the MGBA [123]. In addition, treatment with Ping-wei-san plus may adjust the gut microbiota, modify the biological pathways of metabolites, and influence the presentation of functional pathway proteins, resulting in therapeutic effects on PD [124]. Salidroside (SAL) has been shown to alleviate LPS-induced cognitive impairment, reduce toxic Aβ_1-42_ deposition, and improve gut microbiota and gut barrier integrity in AD [125]. In addition, other agents have been shown to be protective. For example, rifaximin can modulate gut microbial composition, increase gut barrier integrity, and rescue cognitive impairment induced by circadian rhythm disruption [126]. *Rumex japonicus Houtt*. protects dopaminergic neurons by regulating MGBA and mitochondrial function [127]. Morin exerts a neuroprotective effect on PD by enhancing antioxidant defence and anti-inflammatory mechanisms with the involvement of MGBA [128]. More positively, some medicines have entered clinical trials for the treatment of NDs. FLZ, a novel squamosamide, has shown efficacy in many models and is in phase I clinical trials for the treatment of PD [129]. The more advanced sodium oligomannate (GV-971) completed its first phase 3 clinical trial in AD [130, 131].

Dietary modification is an appropriate nutritional strategy to prevent NDs [132]. A fiber-deficient diet can cause cognitive impairment and loss of hippocampal microglia-mediated synapses *via* the gut microbiota and its metabolites [133]. High consumption of chicken and pork protein remarkably enhances levels of systemic inflammatory factors, tau protein, and amyloid precursor protein mRNA in the hippocampus and alters the balance between the glutamatergic system and neurotransmitters, ultimately exacerbating inflammation and disruption of the hippocampal glutamatergic system [134]. Therefore, dietary changes and nutrient supplementation are necessary to improve NDs. For example, a ketogenic diet has been shown to affect neuronal apoptosis, neuroinflammation, and MGBA, supporting this effective treatment approach for NDs [135, 136]. A Mediterranean diet may also have a positive effect on both redox imbalance and inflammatory response, as well as influencing the composition of the microbiota, which may contribute to the improvement of NDs [137]. Furthermore, dietary supplementation of microbiota-accessible carbohydrates may prevent neuroinflammation and cognitive decline by improving MGBA and reducing the risk of developing diet-associated NDs [133, 138]. Long-term β-glucan supplementation can reverse the effects of a high-fat and fiber-deficient diet on MGBA and cognitive function [139]. Meanwhile, sea buckthorn polysaccharide can reverse gut dysbiosis and benefit cognitive dysfunction [140]. Sesamol can improve spatial memory and learning ability in AD mice, ameliorate neuronal injury, and reduce Aβ accumulation [141], and chicoric acid can prevent neuroinflammation and gut inflammation [142]. These unequivocally demonstrate that the advancement of nutraceuticals can be tremendously advantageous to neurological disorders.

Rather than being confined to the singular application of these therapies, employing them synergistically appears to offer enhanced prospects. For instance, probiotics modulate gut-brain interactions and neuroinflammation, while vitamins maintain neuronal health and cognitive function [143]. Therefore, probiotic and vitamin co-supplementation can reduce oxidative stress, inflammation, and gut dysbiosis, which extends the aging trajectory and indirectly delays the onset of AD [143]. Combining the characteristic fatty acid 10-Hydroxydecanoic acid (10-HDAA) in royal jelly and aspirin has a synergistic effect against memory deficit and neuroinflammation [144]. This combinatory group alleviates LPS-induced inflammation in BV-2 cells, synergistically inhibits the overactivation of glial cells, and decreases the levels of pro-inflammatory mediators [144]. Importantly, 10-HDAA can alleviate the adverse effects of aspirin on gastrointestinal injuries and microbiota dysbiosis [144]. Metformin and cyanidin 3-O-galactoside from *Aronia melanocarpa* synergistically prevent neuronal loss, reduce Aβ aggregation, decrease indole, methyl esters, and ketones, and increase SCFAs and alcohols in feces and urine, alleviating cognitive impairment in SAMP8 mice [145]. Citicoline/Coenzyme Q10/Vitamin B3 fixed combination exerts beneficial and synergistic effects in reducing inflammation and oxidation and in stimulating neurotrophin production in neuronal cells, with co-administration being generally more effective than single ingredients [146]. The synergistic nutraceutical combination D_5_L_5_U_5_, containing docosahexaenoic acid, luteolin, and urolithin A, inhibits Aβ_1-42_-induced toxicity by protecting mitochondria against AD, and the effects of D_5_L_5_U_5_ precedes its single constituents [147, 148]. Accordingly, the synergistic use of these therapies is helpful in increasing therapeutic activity and reducing toxicity. However, we still believe that a healthy lifestyle, rather than the use of multiple nutraceuticals, is more likely to reduce the risk of developing NDs (Table **2**).

As mentioned above, these MGBA-targeted therapies are currently aimed at modifying the gut microbiota, and there are many issues that need to be addressed in further investigations. FMT is a novel approach to restoring gut dysbiosis in the treatment of NDs, which alleviates symptoms with minimal or no adverse side effects [34, 149]. The application of probiotics and prebiotics, which can change the microbial partners and their products for the purpose of treating NDs, is a direct intervention in the beneficial microbiota [150]. However, whether exogenous supplementation of these products has limitations in terms of duration, dosage and species of use, and whether supplementation therapy needs to be targeted according to individual variation in the gut microbiota of patients, taking into account the influence of geography, environment, and diet [151, 152], all these issues should be considered in future studies. Naturally, individualized dietary modification, an easily achievable therapy that is also based on gut microbiota, is one of the least costly and least effective approaches for patients with NDs, and its combination with other therapies is also one of the trends in prospective studies [153, 154]. In addition, although some existing medicines targeting NDs are already in clinical trials [129-131], efforts should be made to reduce the side effects of medicines in the future, and it is also essential to advance the identification of other ways or medicines that intervene in disease progression from multiple targets in the MGBA [152, 155]. Overall, there is great potential in focusing on the gut microbiota of MGBA in the study of treatments for NDs, but MGBA-targeted therapy for NDs should be more comprehensive and standardized.

## 7 CURRENT OUTSTANDING CHALLENGES, LIMITATIONS, AND PRESENT STATUS

Currently, a significant portion of studies investigating therapies targeting the MGBA in the context of NDs and its involvement in ND pathogenesis exhibit a limitation in comprehensively simulating the multifaceted etiology. As potential therapeutic agents and dietary modifications transition from animal studies to clinical trials, their beneficial effects often prove to be suboptimal or non-existent, which may be attributed to the models employed to study the MGBA [155]. Existing animal models have limited capacity to faithfully replicate human diseases and are less adept at reproducing the metabolic and inflammatory dysfunctions observed in distant organs such as the gastrointestinal tract [155, 156]. For instance, 6-hydroxydopamine (6-OHDA)-induced Parkinson's disease (PD) models lack independent prodromal features resulting from gut microbiota dysbiosis and gut dysfunction, thereby limiting the construct or predictive validity of these models [157]. Moreover, animal models, such as rodents, differ from humans in terms of high individual variation, evolutionary isolation, and anatomical heterogeneity, thereby imposing constraints on research [158, 159]. Disparities exist in the complexity of neural networks, genetic homology, and reproductive cycles between rodents and humans. While transgenic animals or non-human primate models may offer improved suitability, their development also necessitates additional testing [159]. Furthermore, the emergence of *in vitro* models partially addresses the limitations of animal models. However, their simplicity relative to the intricate *in vivo* conditions restricts their efficacy for MGBA research applications [158, 160, 161]. Hence, the establishment of robust *in vivo* and *in vitro* models serves as the cornerstone for MGBA research and represents a significant hurdle in advancing MGBA-targeted therapies for NDs.

Given the intricate interplay between NDs and the MGBA, future research models should adopt a multi-organ perspective to capture the complexity of these interactions. In addition to incorporating the gut microbiota as a central component, models should encompass various cell types, including gut epithelial, endothelial, stromal, neuronal, and immune cells, to encompass all key players of the MGBA [161]. Notably, the liver, which plays a crucial role in the primary metabolic pathways of carbohydrates, lipids, and proteins, appears to be involved in the MGBA and should be considered for inclusion in relevant models [161, 162]. Moreover, multi-organ models should possess the capability to independently control numerous potential contributing factors, if not all of them, to gain a comprehensive understanding of their individual impacts [163]. Exploring whether dysfunction in one or more peripheral organs occurs in a defined sequence that ultimately constitutes a risk factor for NDs would be highly valuable [163]. Furthermore, the development of multi-organ models will facilitate the design of multi-target directed ligands, which could represent a more rational approach to treating NDs [154].

**Fig. (6):**
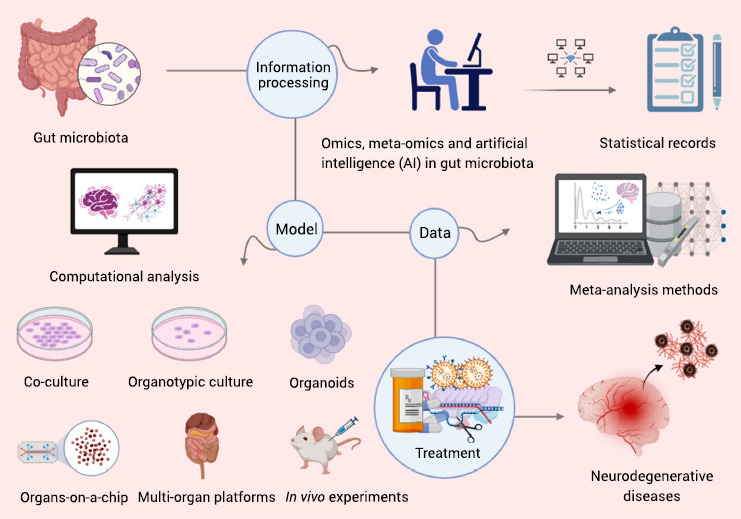
Emerging technologies can be applied to explore the interactions between the microbiota-gut-brain axis (MGBA) and neurodegenerative diseases (NDs). Omics, meta-omics, and artificial intelligence (AI) can analyze and process large amounts of data related to gut microbiota, allowing for statistical analysis and linking it to NDs. This information can serve as a basis for establishing models that combine physiologically relevant conditions with computational analysis. Computational methods can be integrated with co-culture, organotypic culture, and organoids, as well as organs-on-a-chip and multi-organ platforms to further enhance our understanding of MGBA. Additionally, meta-analysis can create a statistical database of clinical trials, providing valuable insights for diagnosing and predicting diseases from multiple dimensions. Ultimately, these emerging technologies hold promise for the development of therapies targeting MGBA in the treatment of NDs.

It is imperative to enhance the implementation of treatments for NDs by integrating emerging technologies into existing approaches (Fig. **6**). For instance, the application of clustered regularly interspaced short palindrome repeats (CRISPR) technology allows for the engineering of therapeutic probiotics [151]. This approach enables selective antimicrobial targeting to regulate the microbiota and the modification and insertion of genes to enhance the effects on NDs [151]. Furthermore, a comprehensive approach leveraging the potential and experimental capabilities of omics and meta-omics sciences holds promise in advancing microbiota research [164]. This approach will elucidate the causal relationship between the gut microbiota and NDs, thereby facilitating the development of novel therapeutic strategies targeting the gut microbiota and its metabolites. The administration of medicines also plays a critical role in treatment outcomes. Nanoparticles have demonstrated effects on both beneficial and pathogenic bacteria in the human gut, and the efficacy of oral nanomedicines can be influenced by factors such as pH and electrolyte concentrations in gastric fluids, as well as the interaction with and permeation through the mucus layer [165]. In this regard, the design of nanocarriers and the incorporation of a propeller system present promising options [165]. Overall, the exploration of therapies for NDs will persist as a prevailing trend and a perpetual challenge to be addressed.

Furthermore, artificial intelligence (AI) holds immense potential in comprehending the gut microbiota alterations associated with NDs and in developing multi-organ models. AI can efficiently process vast amounts of data on the composition and abundance of gut microbiota and establish correlations with disease states. Leveraging this information, models can be constructed to identify specific gut microbiota types and analyze their roles within the MGBA. For instance, computational methods can be combined with co-culture, organotypic culture, organoids, and organs-on-a-chip and integrated with multi-organ platforms like the MINERVA project [158, 166]. Consequently, the integration of physiologically relevant conditions with AI presents a viable approach for investigating the MGBA in future studies. These models can also be employed to assess the effectiveness of treatments in modulating the gut microbiota and to identify the specific gut microbiota that impact acute or chronic diseases. Additionally, meta-analysis techniques can be applied to establish a statistical database of studies, systematically elucidating the interaction between the MGBA and NDs [166]. Therefore, it is plausible that AI will emerge as the primary tool for studying NDs in the future.

## CONCLUDING REMARKS AND FUTURE PERSPECTIVES

**Fig. (7):**
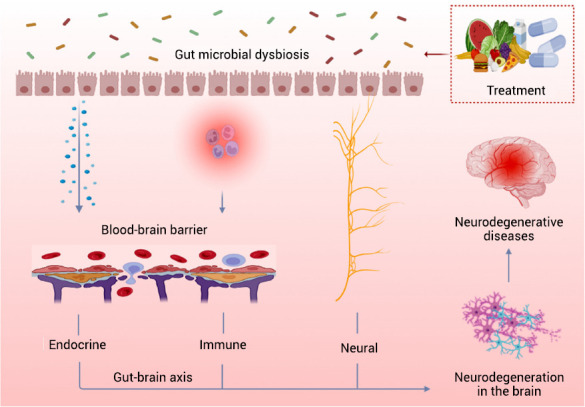
Microbiota-gut-brain axis (MGBA) plays a significant role in the pathogenesis of neurodegenerative diseases (NDs). Dysbiosis of the gut microbiota can lead to gut leakage and trigger inflammatory responses. Various molecules in the gut can reach the brain through the gut-brain axis, including neural, immune, and endocrine routes. This can result in increased permeability of the blood-brain barrier and subsequent neurodegeneration, ultimately leading to the development of NDs. Accordingly, treatments targeting the MGBA have shown promise in improving the symptoms of NDs.

By placing a strong focus on gastrointestinal alterations observed in individuals with NDs, it becomes evident that dysbiosis of the gut microbiota possesses the potential to initiate structural disruptions within the gut barrier. Consequently, this leads to compromised gut functionality and triggers inflammation not only within the gastrointestinal tract but also systemically. Notably, alterations in cell populations within the BBB serve as indicative markers of its compromised integrity, resulting in dysfunctional processes and neuroinflammatory responses. Collectively, these changes occurring simultaneously in the gut and brain contribute to the progression of NDs. It is important to recognize that the two components are intricately interconnected, with the MGBA serving as a vital bridge between them. The gut microbiota and its metabolites play a role in the multifaceted regulation of NDs through neural, endocrine, and immune pathways, encompassing the central nervous system (CNS), autonomic nervous system, enteric nervous system, hypothalamic-pituitary-adrenal axis, immune system, and endocrine system. Consequently, we strongly believe that alterations in gut microbiota may serve as pivotal factors in modulating the development of NDs, with fecal FMT emerging as one potential intervention. In addition to FMT, other interventions include exogenous supplementation with probiotics or prebiotics, herbal extracts, medications, and dietary modifications (Fig. **7**).

In this work, we have compiled the results of comprehensive experiments to elucidate the association between NDs and various changes induced by the gut microbiota and its metabolites. However, we have refrained from providing specific details regarding which gut microbiota or metabolites exert their effects in particular locations. This omission stems from the vast diversity and abundance of these entities among individuals, potentially disrupting the gut barrier, the BBB, and potentially generating inflammatory factors. Additionally, they may accumulate in the brain as cumulative substances that contribute to disease initiation. Furthermore, the dysbiosis of the gut microbiota has not been extensively discussed, and we have only touched upon the different types and degrees of dysbiosis observed in various NDs, without exploring whether each disease exhibits distinct alterations in specific gut microbiota and their metabolites. Moreover, the MGBA serves as a comprehensive communication route between the gut and brain, and our focus has primarily been on elucidating the multiple pathways through which the gut microbiota and its metabolites influence the brain, ultimately leading to the development of NDs.

While there is a burgeoning body of clinical and biomedical evidence linking gut microbiota to NDs, future investigations require more rigorous and discerning methodologies to establish the relationship definitively. The current studies predominantly rely on post-onset findings of NDs, lacking comprehensive pre-onset monitoring data. Consequently, long-term studies are imperative to ascertain the extent of BBB and gut barrier disruptions, as well as the dysbiosis of the gut microbiota as potential triggers for NDs. Three primary pathways of the MGBA have been identified as being implicated in this context. However, further elucidation is necessary to better comprehend the precise mechanisms through which distinct gut microbiota traverse these pathways. Specifically, a more comprehensive understanding of the bidirectional regulation between the gut microbiota and the CNS necessitates additional fundamental and clinical research, focusing on the specific molecular and biological mechanisms involved. Additionally, it is crucial to explore the existence of alternative communication pathways in the forthcoming years. Notably, the composition and abundance of gut microbiota associated with NDs are anticipated to undergo continuous evolution, facilitating the identification and classification of substances directly associated with these diseases.

## AUTHORS’ CONTRIBUTIONS

TN and LY wrote the draft and prepared the figures. YD searched the references and revised the manuscript in consultation with QW and WW. EN, FT, and KK revised the manuscript. QW and WW contributed to the article design and the data analysis and corresponding.

## Figures and Tables

**Table 1:** Changes of gut microbiota in patients with NDs or animals.

**Diseases**	**Model**	**Changes in Gut Microbiota and its Metabolites**	**References**
AD	Preclinical AD individuals	*Bacteroides intestinalis*, *Bacteroides caccae*, *Methanobrevibacter smithii *are found in preclinical AD. *Alistipes*, *Barnesiella*, and *Odoribacter *are found in symptomatic individuals with AD. No significant difference in the total *Bacteroidetes* to *Firmicutes* ratio between healthy and preclinical AD individuals.	[167]
AD	3xTg-AD mice	*Turicibacter *and *Akkermansia muciniphila* are enriched at early time points prior to the development of pathology, while *Bacteroides acidifaciens* and *Prevotella *are enriched at later time points. The composition of the gut microbiota becomes more similar between strains with aging.	[168]
AD	App^swe^/PS1^ΔE9^ transgenic mice	Increased *Bacteroidetes* and *Firmicutes*, decreased *Proteobacteria*, fluctuating *Verrucomicrobia.* Gut microbiota diversity decreases and gut microbiota patterns vary.	[169]
AD	3xTg-AD mice	The *Firmicutes/Bacteroidetes *ratio increases, the *Candidatus Saccharibacteria phylum* and *Proteobacteria* increase. Capric, benzeneacetic acid, levulinic, 4-hydroxybenzeneethanol, and hydrocinnamic acid are up-regulated, while hexadecenoic acid, octadecanoic acid, and 3-phenylpropanol are under-represented.	[170]
AD	APP/PS1 mice	The ratio of the abundance of *Firmicutes* to *Bacteroidetes* shifts with age. Inflammation-associated *Escherichia-Shigella*, *Desulfovibrio, Akkermansia*, and *Blautia *increase. Differences in abundance and diversity of gut microbiota increase with age.	[171]
PD	PD patients	*Actinobacteria*, *Proteobacteria*, and *Verrucomicrobia *enrich, *Bacteroidetes*, *Cyanobacteria*, and members of the *Firmicutes* decrease.	[172]
PD	A53T transgenic monkeys	3-Methylindole and myristic acid decrease, glyceric acid, L-aspartic acid, and p-hydroxyphenylacetic acid increase. *Acidobacteria*, *Sybergistetes*, and *Nitrospirae* increase, *Bacteroidetes* decrease, *Proteobacteria* and *Actinobacteria* are not significantly different.	[27]
PD	PD patients and mice	Acetate, butyrate, and propionate are significantly downregulated, especially propionate. Propionate exerts beneficial effects on intestinal epithelial barrier function and improves motor behavior in PD mice through the proteirrserinc-threonine kinases pathway.	[28]
PD	PD patients	Butyrate, tyrosine, and phenylalanine changes are highly associated with PD. They may be involved in the apoptosis signaling pathway, inflammation mediated by chemokine and cytokine signaling pathway, dopamine receptor-mediated signaling pathway, and oxidative stress response.	[173]
PD	PD patients	*Desulfovibrio *increases, and its concentration correlates with the severity of PD. *Desulfovibrio* produces hydrogen sulfide and LPS, and several strains synthesize magnetite, which can induce the oligomerization and aggregation of the a-synuclein protein.	[174]
HD	Drosophila Model	Gut dysbiosis is characterized by elevated levels of total bacteria, and *Gram-negative Acetobacter* and *E. coli* are modifiers of HD pathogenesis.	[175]
HD	R6/1 mice	*Bacteroidetes* phylum increases, *Firmicutes* phylum decreases proportionally. *Bacteroidetes* is the most abundant phylum in HD mice, followed by *Firmicutes*, *Actinobacteria*, *Proteobacteria,* and the remaining phyla. Changes in the gut microbiota vary by sex.	[29]
HD	R6/2 mice	*Bacteroidetes (Gram−)* increase and *Firmicutes (Gram+) *decrease. FD4 uptake into the blood circulation, a marker for increased gut permeability, varies with the abundance of predominantly Gram-negative members, including the Proteobacteria phylum and the *Parabacteroides* genus.	[48]
ALS	ALS patients	*Roseburiaintestinalis, Eubacteriumrectale, Bilophila, Clostridiaceae bacterium JC118, Coprobacterfastidiosus, Eubacteriumeligens, *and *Ruminococcussp 5139 BFAA* decrease. *Escherichia* and *Streptococcus salivarius* increase.	[176]
ALS	ALS patients	*Bacteroidetes* at the phylum level and several microbes at the genus level are up-regulated, *Firmicutes* at the phylum level, and *Megamonas* at the genus level are down-regulated.	[30]
MS	MS patients	*Parabacteroides*,* Bacteroides stercoris*,* Bacteroides coprocola*,* Bacteroides coprophilus*,* Provotella copri*,* Clostridia*,* Sutturella*,* Haemophilus*,* Aldercreutzia and Collinella *decrease. *Blautista*, *Dorea*, *Streptococcus thermophilus*, *Pedobacteria*, *Flavobacterium*, *Pseudomonas* and *Mycoplana* and *Eggerthella lenta* increase.	[177]
MS	C57BL/6 mice	*Actinobacteria, Bacteroidetes, Cyanobacteria, Deferribacteres, Firmicutes, Proteobacteria, Spirochaetes, *and *Tenericutes* fluctuate, and *Verrucomicrobia *decrease. Statistical differences are detected from the phylum to the genus level.	[178]

**Abbreviations**: AD: Alzheimer's disease; PD: Parkinson's disease; HD: Huntington's disease; ALS: Amyotrophic lateral sclerosis; MS: Multiple sclerosis.

**Table 2:** Summary of the microbiota-gut-brain-targeted therapy in NDs.

**Product/Diet**	**Model**	**Time**	**Dose**	**Function**	**References**
*L. plantartun* DR7 (DR7)	Drosophila melanogaster AD model	14-15 days	1×10^11^ CFU/mL	Improve gut microbiota profile and alleviate neurodegeneration in the eye.	[179]
*L. rhamnosus* UBLR-58	Scopolamine-induced AD model	10 days	1×10^6^ CFU/day	As a curcumin adjuvant (potentiating the anti-Alzheimer activity of curcumin), it improves memory and cognitive functions.	[180]
*Bacillus coagulans* JA845	D-gal/AlCl3-induced AD model	10 weeks	1×10^9^ CFU/day	Prevent cognitive decline, attenuate hippocampal lesions, protect neuronal integrity, and alleviate Aβ deposition and hyperphosphorylated tau.	[181]
*Acidophilus*	AD model	4 weeks	12×10^8^ CFU/mL	Alleviate the damage associated with learning and memory by reducing mitochondrial dysfunction.	[182]
MG136-pMG36e-GLP-1	AD and PD model	7 days	1×10^9^ CFU/day	Reduce memory impairment and motor dysfunction, reduce Enterococcus Proteus, and increase Akkermansiamuciniphila.	[183]
The mixture of *L. acidophilus*, *B. bifidum*, *L. reuteri*, and* L. fermentum*	6-OHDA-induced PD model	14 days	Products	Improve rotational behavior, cognitive function, lipid peroxidation, and neuronal damage.	[184]
Oligofructose + probiotic	Pre-frail individuals	24 weeks	6 g+6 g/day	Reduced depressive symptoms and improved cognitive functioning.	[118]
*Lactobacillus paracasei HII01, Bifidobacterium animalis subsp,* Lactis, galacto-oligosaccharides, oligofructose	Thai volunteers	12 weeks	10^10^ CFU+10 g	Reduce negative emotions by modulating HPA axis activation and IL-10, IgA, and LPS production.	[119]
Butyrate	Obesity model	15 weeks	5% w/w in diet	Counteract BDNF reductions, neuronal spine loss and cognitive impairment to alleviate neurodegeneration.	[122]
SLAB51	6-OHDA-induced PD model	5 weeks	270 µl, 0.5 g/mL	Increase neuroprotective protein levels, decrease neuronal death proteins, protect dopaminergic neurons, and improve behavioral impairments.	[185]
*L. rhamnosus* GG, *B. animalislactis*, and *L. acidophilus*	MPTP or rotenone-induced PD model	30 days	2×10^6^ CFU/day	Increase butyrate rescue the nigral dopaminergic neurons.	[186]
*L. lactis *MG1363-pMG36e-GLP-1	MPTP-induced PD model	7 days	1×10^9^ CFU/day	Reverse dysbiosis, modulate oxidative stress and inhibit ferroptosis.	[187]
VSL#3	PD model	6 weeks	4 × 10^9 ^CFU/dose	Protect dopaminergic neurons.	[188]
*L. plantarum *PS128	MPTP-induced PD model	28 days	1×10^10^ CFU/mL	Alleviate motor deficits, corticosterone elevation, and neuronal death, as well as attenuate oxidative stress and neuroinflammation.	[189]
Yeast β-glucans	Aβ-induced AD model	4 weeks	100 mg/kg BW	Produce SCFAs and shape some beneficial and inflammatory bacteria.	[113]
Mannan oligosaccharide	5xFAD AD model	8 weeks	0.12% w/v in the drinking water	Improve cognitive and memory function, suppress neuroinflammation, alleviate HPA axis disorders, prevent barrier damage, and reconstruct gut microbiota.	[114]
Neoagarotetraose	C57BL/6J mice and APP/PS1 double transgenic mice	12 months	1000 mg/kg BW/day	Reshape gut microbiota, enhance gut barrier integrity, suppress inflammation, and reduce brain damage and cognitive impairment.	[115]
Ping-wei-san plus herbal decoction	PD model	90 days	6-7 g/day	Modulate gut microbiota, alter biological pathways of metabolites, and influence expression of functional pathway proteins.	[124]
Herbal medicine formula Jiao-tai-wan	Sleep-deprived model	8 weeks	1.1 or 2.2 g/kg/day	Inhibit inflammation of the gut-brain axis and attenuate systemic inflammation and cognitive impairment.	[123]
Morin	Rotenone-induced PD model	28 days	5, 20 or 80 mg/kg	Reverse behavioral deficits by enhancing antioxidant defenses and anti-inflammatory mechanisms.	[128]
Salidroside	SAMP8 model	3 months	50 mg/kg/day	Alleviate cognitive impairment, reduce Aβ1-42 deposition, improve gut barrier integrity and gut microbiota, and reduce pro-inflammatory cytokines.	[125]
GV-971	AD model	36 weeks	450 mg, b.i.d.	Suppress gut dysbiosis, harness neuroinflammation, and reverse cognitive impairment.	[130, 131]
Rifaximin	CRD-induced model	1 weeks	250 mg/kg/day	Modulate gut microbial composition, increase gut barrier integrity, suppress inflammatory responses, and rescue cognitive impairment.	[126]
Novel compound FLZ	Rotenone-induced PD model	2 weeks	75 mg/kg/day	Reverse PD-related microbiota alterations, attenuate gut barrier destruction, restore blood-brain barrier structure, and suppress inflammation.	[129]
Rumex japonicus Houtt.	MPTP-induced PD model	14 days	50 or 100 mg/kg/day	Inhibit the disruption of the tight junction barrier and alleviate inflammation and dopaminergic neuronal death.	[127]
Ketogenic diet	NDs	days or weeks	diet	This leads to a metabolic shift to ketosis, which affects mitochondrial function, oxidative stress, neuronal apoptosis, neuroinflammation, and the MGBA.	[135]
Mediterranean diet	PD model	weeks or years	diet	It affects both the redox imbalance and the inflammatory response and has an impact on the composition of the microbiota.	[137]
Microbiota-accessible carbohydrates	Diet-induced obese mice	15 weeks	316 g/kg from fat; LabDiet 5010 634 g/kg	Prevent neuroinflammation and cognitive decline with MGBA and reduce the risk of developing diet-related NDs.	[133, 138]
Long-term β-glucan supplementation	HFFD-induced cognitive impairment	15 weeks	Oat β-glucan derived from OatWell^TM^ oat bran	Improve the synaptic ultrastructure, reverse gut barrier dysfunction, attenuate bacterial endotoxin translocation, and reverse microbiota alteration.	[139]
SBP	HFD mice	12 weeks	High-fat-diet chow containing 0.1% SBP (w/w)	Alleviate behavioral disorders, alleviate gut barrier damage, inflammatory responses, and lipopolysaccharide entry into blood circulation.	[140]
Sesamol	AD model	8 weeks	0.075 % (w/w) in the standard feed	Protect the synaptic ultrastructure, inhibit neuroinflammatory responses, inhibit the overactivated microglia, reshape gut microbiota, and protect the gut barrier.	[141]
Chicoric acid	MPTP-induced PD model	12 days	60 mg/kg	Reduce microbial dysbiosis, promote colonic epithelial integrity, restore normal SCFA production, and reduce pro-inflammatory cytokines.	[142]
10-hydroxydecanoic acid + aspirin	BV-2 microglia cells, male C57BL/6J mice	pre-treat 1 hour	Variation	Alleviate LPS-induced inflammation, inhibit glial cells overactivation, and decrease pro-inflammatory mediators	[144]
Metformin + cyanidin 3-O-galactoside	SAMP8 mice	8 weeks	100 mg/kg, 25 mg/kg	Prevent neuronal loss, reduce Aβ aggregation, and decrease indole, methyl esters, ketones, and increase SCFA alcohols in feces and urine.	[145]
Citicoline + Coenzyme Q10 + Vitamin B3	HypoE22 cells, C57BL6 mice	Different time	1 nM-10 μM	Reducing inflammation and oxidation stimulates neurotrophin production in neuronal cells.	[146]
Docosahexaenoic acid + luteolin + urolithin A	BE (2)-M17 cells	Passage < 30	5 + 5 + 5 μM	Inhibit Aβ1-42-induced toxicity by protecting mitochondria and then against AD.	[147, 148]

**Abbreviations**: *B.*: *Bifidobacterium*; CRD: circadian rhythm disruption; GV-971: Sodium oligomannate; HFD: high-fat diet; HFFD: high-fat and fiber-deficient diet; *L.*:*Lactobacillus*; MPTP: 1-methyl-4-phenyl-1,2,3,6-tetrahydropyridine; SAMP8: Senescence-Accelerated Mouse Prone; SBP: sea buckthorn polysaccharide; SCFAs: short-chain fatty acids; SLAB51: *Streptococcus thermophilus, B. longum, B. breve, B. infantis, L. acidophilus, L. plantarum, L. paracasei, L. delbrueckii subsp. bulgaricus and L. brevis*; 6-OHDA: 6-hydroxydopamine.

## LIST OF ABBREVIATIONS

AD: Alzheimer's Disease
AI: Artificial Intelligence
ANS: Autonomic Nervous System
AβO: Amyloid-β Oligomer
BBB: Blood-brain Barrier
CNS: Central Nervous System
ENS: Enteric Nervous System
FMT: Fecal Microbiota Transplantation
GABA: Gamma-aminobutyric Acid
HD: Huntington's Disease
HPA: Hypothalamic-pituitary-adrenal
5-HT: 5-hydroxytryptamine
IL-1 β: Interleukin-1 β
LDL: Low-density Lipoprotein
LPS: Lipopolysaccharide
MGBA: Microbiota-gut-brain Axis
MOS: Mannan Oligosaccharide
MS: Multiple Sclerosis
NDs: Neurodegenerative diseases
PD: Parkinson's Disease
SAL: Salidroside
SCFAs: Short-chain Fatty Acids
TMAs: Trimethylamines
TNF-α: Tumor Necrosis Factor-alpha
